# A novel SERPING1 splice-site variant (c.1029 + 2T > A) causing hereditary angioedema type I: functional characterization and clinical analysis

**DOI:** 10.1186/s13023-026-04421-3

**Published:** 2026-06-02

**Authors:** Weili Guo, Xu Yang, Wenjin Du, Xianghua Lin, Wenchao Zhang, Qiuxing Zhang, Zhaoji Meng, Siqin Wang, Shixiu Liao, Zhenglong Guo

**Affiliations:** Henan Provincial People’s Hospital, People’s Hospital of Zhengzhou University, Zhengzhou, 450003 China

**Keywords:** Hereditary angioedema, *SERPING1*, C1 esterase inhibitor, Splice-site variant, Exon skipping, Minigene assay, Lanadelumab, Long-term prophylaxis

## Abstract

**Background:**

Hereditary angioedema (HAE) is a rare autosomal dominant disorder predominantly caused by mutations in the *SERPING1* gene, which encodes C1 esterase inhibitor (C1-INH). HAE is characterized by recurrent self-limiting episodes of subcutaneous and submucosal edema that can be life-threatening when the upper airway is involved. Diagnostic delays and misdiagnoses are common due to the clinical overlap with allergic and histamine-mediated angioedema. Identification and functional characterization of novel *SERPING1* variants is essential for refining the mutational spectrum of this disease, enhancing molecular diagnostics, and guiding individualized therapeutic strategies.

**Methods:**

A 31-year-old female with a ten-year history of recurrent angioedema was diagnosed with HAE type I based on reduced serum C4, C1-INH antigen levels, and C1-INH functional activity. Sanger sequencing of the *SERPING1* gene was performed to identify the causative variant. Functional characterization of the identified splice-site variant was conducted using reverse transcription-PCR (RT-PCR) on patient-derived peripheral blood RNA and an in vitro minigene splicing assay in HEK293T cells. A retrospective analysis of seven HAE patients was performed, including clinical characterization, laboratory profiling, genetic analysis, and treatment outcomes.

**Results:**

A novel heterozygous splice-site variant, c.1029 + 2T > A, was identified at the canonical splice donor site of intron 6 of *SERPING1*. RT-PCR revealed reduced wild-type transcript expression and an aberrant transcript resulting from complete exon 6 skipping (c.890_1029del), predicted to cause a frameshift and premature termination codon (p.Ala297Glyfs*25). The minigene assay confirmed that the mutant construct produced no detectable canonical transcript; instead, three aberrant splice isoforms were identified, with exon 6 skipping as the predominant event. Long-term prophylactic treatment with Lanadelumab (300 mg subcutaneously every two weeks) achieved zero breakthrough attacks over a six-month follow-up period. In the retrospective cohort of seven patients, six were classified as type I and one as type II HAE. Seven distinct variants of the *SERPING1* gene were identified. Among these patients, five received long-term prophylaxis with Lanadelumab, which resulted in significant reductions in the frequency of attacks.

**Conclusion:**

We identified and functionally characterized a novel *SERPING1* splice-site variant, c.1029 + 2T > A, which disrupts canonical mRNA splicing, primarily through exon 6 skipping, leading to C1-INH haploinsufficiency and type I HAE. Our retrospective analysis expands the mutational spectrum of *SERPING1* and demonstrates the clinical efficacy of Lanadelumab as a long-term prophylactic therapy. These findings underscore the importance of integrating genetic testing and functional validation in the diagnosis and management of HAE.

**Supplementary Information:**

The online version contains supplementary material available at 10.1186/s13023-026-04421-3.

## Methods

### *SERPING1 g*ene sequencing

Genomic DNA was extracted from peripheral blood samples of patients using standard protocols. The entire coding region and exon-intron boundaries of the *SERPING1* gene (NM_000062.3) were amplified by polymerase chain reaction (PCR) using previously established primer sets [1]. Next-generation sequencing was performed to identify genetic variants. Variant calling and annotation were conducted using bioinformatics pipelines, and pathogenicity was assessed according to the American College of Medical Genetics and Genomics (ACMG) guidelines. Candidate pathogenic variants were confirmed by Sanger sequencing.

### Patient-derived mRNA splicing analysis

Peripheral blood mononuclear cells (PBMCs) were isolated from a patient and an age-matched healthy control. Total RNA was extracted from the PBMCs using TRIzol reagent (Invitrogen, CA, USA) in accordance with the manufacturer’s instructions. The purity and concentration of the RNA were determined using a Nanodrop spectrophotometer (Thermo, MA, USA). First-strand cDNA synthesis was performed using HiScript IV All-in-One Ultra RT SuperMix (Vazyme, Nanjing, China). The *SERPING1* transcript, encompassing exons 5–7, was amplified via PCR using the following primers: Forward: 5’-TTGTGAATGCCTCTCGGACC-3’ and Reverse: 5’-GCTGGTCGTCACTTTGATGC-3’. The PCR products were analyzed through agarose gel electrophoresis, and we sequenced aberrant bands by TA-cloning and Sanger sequencing to characterize the splicing pattern (Tsingke Biotechnology Co., Ltd., Beijing, China).

### Minigene splicing assay

To validate the functional impact of the splice-site variant, an in vitro minigene splicing assay was performed. Genomic fragments encompassing *SERPING1* exons 5–7 and their flanking intronic sequences were PCR-amplified from patient genomic DNA and cloned into the pMini-CopGFP vector using BamHI and XhoI restriction sites. Both wild-type and mutant (c.1029 + 2T > A) minigene constructs were generated. The constructs were verified by Sanger sequencing.

HEK293T cells were cultured in Dulbecco’s Modified Eagle Medium (DMEM) supplemented with 10% fetal bovine serum (FBS) and maintained at 37 °C in a humidified atmosphere with 5% CO₂. Cells were seeded in 6-well plates and transfected at 70–80% confluence using Lipofectamine 3000 (Invitrogen) according to the manufacturer’s protocol. At 48 h post-transfection, total RNA was extracted using TRIzol reagent, and cDNA was synthesized by reverse transcription. The minigene-derived transcripts were amplified by PCR using vector-specific primers flanking the cloned insert. PCR products were analyzed by agarose gel electrophoresis to assess fragment size, and individual bands were gel-purified, cloned, and sequenced by Sanger sequencing to determine the precise splicing outcomes.

### Laboratory measurements

Serum C4 concentration was quantified by nephelometry. C1-INH antigenic concentration was measured by nephelometry. C1-INH functional activity was determined by enzyme-linked immunosorbent assay (ELISA). Reference ranges: C4, 0.10–0.40 g/L; C1-INH concentration, 0.21–0.39 g/L or 81.46–291.29 µg/mL; C1-INH functional activity, ≥ 68.0% (normal), ≤ 50% (abnormal).

### Retrospective clinical case review

Patients diagnosed with HAE who presented to our institution were identified through the electronic medical record system and included in this retrospective analysis. Clinical data, including demographic information, family history, clinical manifestations, laboratory results, genetic findings, and treatment outcomes, were extracted from medical records. Follow-up information regarding treatment efficacy and disease control was obtained through telephone interviews and outpatient visits.

## Background

Hereditary angioedema (HAE) is a rare autosomal dominant disorder with an estimated prevalence of 1 in 50,000. It is characterized by recurrent episodes of self-limiting but potentially life-threatening edema affecting the skin, gastrointestinal tract, and upper respiratory mucosa [2]. Unlike histamine-mediated angioedema, HAE episodes are typically non-pruritic, do not respond to antihistamines or corticosteroids, and can persist for 48 to 120 h before resolving spontaneously [3]. Laryngeal edema, although less frequent than peripheral or abdominal attacks, represents the most dangerous clinical manifestation and accounts for the majority of HAE-related mortality, with historical fatality rates as high as 30% in undiagnosed or untreated patients [4].

The pathophysiology of HAE is primarily linked to a deficiency or dysfunction of C1 esterase inhibitor (C1-INH), a serine protease inhibitor (serpin) encoded by the *SERPING1* gene located on chromosome 11q12.1 [5]. C1-INH regulates multiple proteolytic cascades, including the classical and lectin complement pathways, the contact activation (kallikrein-kinin) system, the coagulation cascade, and the fibrinolytic pathway [6]. Deficiency or dysfunction of C1-INH leads to uncontrolled activation of plasma kallikrein, resulting in excessive cleavage of high-molecular-weight kininogen (HMWK) and the consequent overproduction of bradykinin, a potent vasoactive peptide. Bradykinin binds to the bradykinin B2 receptor on endothelial cells, increasing vascular permeability. This produces the characteristic subcutaneous and submucosal edema observed in HAE.

HAE is classified into three principal subtypes based on the underlying molecular defect [3]. HAE Type I (HAE-1), which accounts for approximately 85% of all cases, is caused by a quantitative deficiency of C1-INH protein, resulting from mutations that reduce C1-INH synthesis, promote intracellular retention, or lead to the secretion of unstable protein. HAE Type II (HAE-2), comprising approximately 15% of cases, is characterized by normal or elevated C1-INH antigenic levels but reduced functional activity, typically resulting from missense mutations affecting the reactive center loop (RCL) or other critical functional domains of the C1-INH protein. A third subtype, HAE with normal C1-INH (HAE-nC1-INH), has been increasingly recognized and is associated with mutations in other genes, including *F12* (coagulation factor XII), *PLG* (plasminogen), *ANGPT1* (angiopoietin-1), *KNG1* (kininogen-1), *MYOF* (myoferlin), and *HS3ST6* (heparan sulfate-glucosamine 3-sulfotransferase 6) [7, 8].

The *SERPING1* gene spans approximately 17 kb and comprises eight exons encoding a 500-amino acid protein, including a 22-amino acid signal peptide. Over 900 pathogenic or likely pathogenic variants have been identified in *SERPING1*, distributed throughout the gene and encompassing a wide spectrum of mutation types, including missense, nonsense, small insertions and deletions (indels), splice-site alterations, large genomic deletions and duplications, and complex rearrangements. Notably, approximately 15–20% of *SERPING1* mutations arise de novo, which explains the absence of a family history in a substantial proportion of HAE patients [9–11]. The mutational heterogeneity of *SERPING1* poses significant challenges for molecular diagnosis and highlights the importance of comprehensive genetic analysis and functional validation of novel variants.

The diagnosis of HAE remains challenging due to its clinical heterogeneity, episodic nature, and phenotypic overlap with other forms of angioedema. The mean diagnostic delay ranges from 8 to 16 years in various populations, during which patients may be misdiagnosed with allergic angioedema, urticaria, or surgical emergencies such as acute appendicitis in abdominal attacks [12, 13]. Current diagnostic criteria for HAE-1 and HAE-2 are based on clinical presentation in conjunction with laboratory assessment of complement C4 levels, C1-INH antigen concentration, and C1-INH functional activity. Genetic testing of *SERPING1* provides definitive molecular confirmation and is particularly valuable in ambiguous cases, for presymptomatic identification of affected family members, and for establishing genotype-phenotype correlations [14, 15].

The therapeutic landscape for HAE has evolved significantly over the past two decades [16]. Acute attacks can be treated with on-demand therapies, including plasma-derived or recombinant C1-INH concentrate, the bradykinin B2 receptor antagonist icatibant, and the kallikrein inhibitor ecallantide. For long-term prophylaxis (LTP), Lanadelumab, a fully human monoclonal antibody targeting plasma kallikrein, has demonstrated robust efficacy in reducing the frequency and severity of HAE attacks. The pivotal HELP trial and subsequent real-world studies have shown that Lanadelumab at 300 mg every two weeks can achieve near-complete attack suppression in a substantial proportion of patients, with a favorable safety profile [17, 18]. Other emerging prophylactic strategies include berotralstat [19], an oral kallikrein inhibitor, and gene therapy approaches currently in clinical development [20].

Despite considerable progress in HAE genetics and therapeutics, the identification and functional characterization of novel *SERPING1* variants remain essential for expanding the mutational spectrum, facilitating precise molecular diagnosis, and enabling personalized therapeutic decision-making. In this study, we report the identification and comprehensive functional characterization of a novel *SERPING1* splice-site variant, c.1029 + 2T > A, in a patient with type I HAE, using both patient-derived transcriptional analysis and an in vitro minigene splicing assay. Additionally, we present a retrospective analysis of seven HAE cases from a single center, encompassing clinical features, laboratory profiles, genetic findings, and treatment outcomes, with the aim of providing a broader perspective on the clinical and molecular landscape of HAE in our population.

## Results

### Case presentation

A 31-year-old woman was admitted to the Department of Allergy at our institution in 2025, presenting with a decade-long history of recurrent angioedema of unknown origin. Her clinical history was characterized by episodic non-pitting edema occurring at various anatomical locations, predominantly affecting the extremities and buttocks. Each episode was localized to a single site and resolved spontaneously within approximately four days. Initially, these episodes occurred two to three times annually, primarily between April and May. However, in the four months before presentation, episodes increased to more than once monthly. The patient had previously been misdiagnosed with endocrine-related edema, and antihistamine treatment had not resulted in any clinical improvement. She reported no family history of angioedema.

**Fig. 1:**
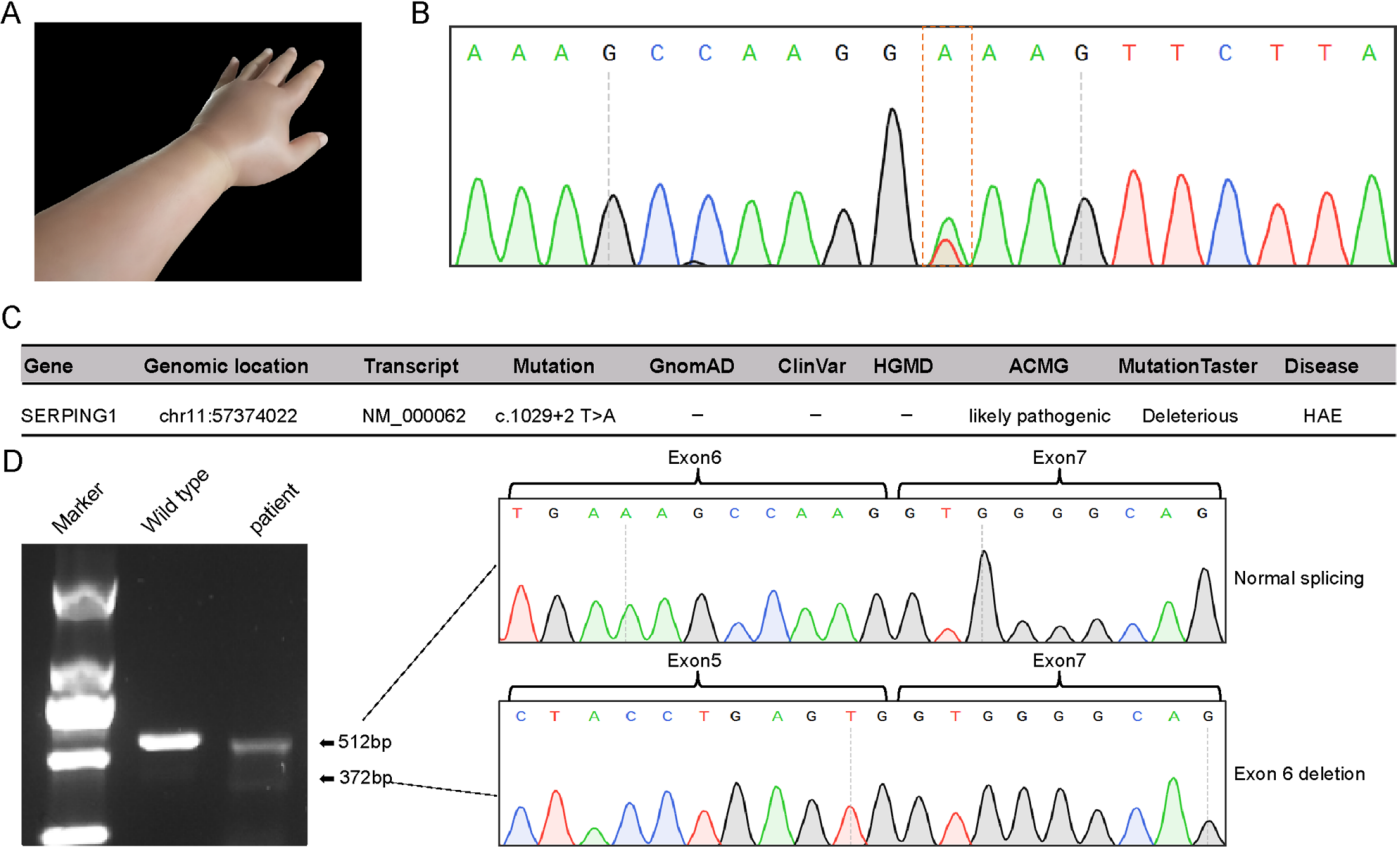
Identification and functional characterization of the *SERPING1* c.1029 + 2T > A splice-site variant. (**A**) Clinical presentation showing left upper limb edema in the proband. (**B**) Sanger sequencing chromatogram confirming the heterozygous c.1029 + 2T > A variant. (**C**) Genomic location and pathogenicity classification of the c.1029 + 2T > A variant according to ACMG guidelines and database annotations. (**D**) RT-PCR analysis of patient-derived peripheral blood mRNA demonstrating aberrant splicing

Upon admission, the patient’s vital signs were stable, and a physical examination revealed mild cutaneous edema in the left upper extremity (Fig. 1A). Laboratory investigations revealed a normal complete blood count, with serum C1q levels and allergen-specific immunoglobulin E (sIgE) concentrations within normal reference ranges. However, the D-dimer level was elevated at 1.13 µg/mL (reference range: 0–0.55 µg/mL). Complement profiling indicated a serum C4 level of 0.08 g/L (reference range: 0.1–0.4 g/L), a C1 esterase inhibitor (C1-INH) antigenic concentration of 0.05 g/L (reference range: 0.21–0.39 g/L), and a C1-INH functional activity of 18.2% (values ≤ 50% are considered abnormal). The reduced C4, C1-INH antigen, and C1-INH functional activity met diagnostic criteria for type I Hereditary Angioedema (HAE).

For molecular diagnosis, we extracted genomic DNA from peripheral blood samples, and the coding region of the *SERPING1* gene (NM_000062) was amplified using polymerase chain reaction (PCR) followed by analysis through Sanger sequencing. A heterozygous variant, c.1029 + 2T > A, was identified at the canonical splice donor site of intron 6. This variant was predicted to disrupt normal mRNA splicing (Fig. 1B). This variant has not been previously reported in the ClinVar database (https://www.ncbi.nlm.nih.gov/clinvar/) or the Human Gene Mutation Database (HGMD; http://www.hgmd.cf.ac.uk/), and it is absent from the Genome Aggregation Database (gnomAD). According to ACMG guidelines, this variant has been classified as likely pathogenic, with evidence codes PVS1 and PM2_Supporting (Fig. 1C).

### Functional characterization of the c.1029 + 2T > A variant

To elucidate the functional impact, we conducted a transcriptional analysis using material derived from the patient. Total RNA was extracted from the peripheral blood of the proband and an age-matched healthy female control. Reverse transcription PCR (RT-PCR) was employed to amplify the *SERPING1* transcript encompassing exons 5 through 7, and the resulting products were analyzed via agarose gel electrophoresis. The healthy control showed a single amplicon of expected size, whereas the patient sample showed a reduced wild-type band and a smaller aberrant band. Sanger sequencing of cloned amplicons confirmed that the upper band (512 bp) corresponded to the canonically spliced transcript, while the lower band (372 bp) resulted from the complete skipping of exon 6 (c.890_1029del) (Fig. 1D). This deletion induces a frameshift and premature termination codon (p.Ala297Glyfs*25) and resulting in a truncated 320-amino-acid protein versus the full-length 500-amino-acid C1-INH.

**Fig. 2:**
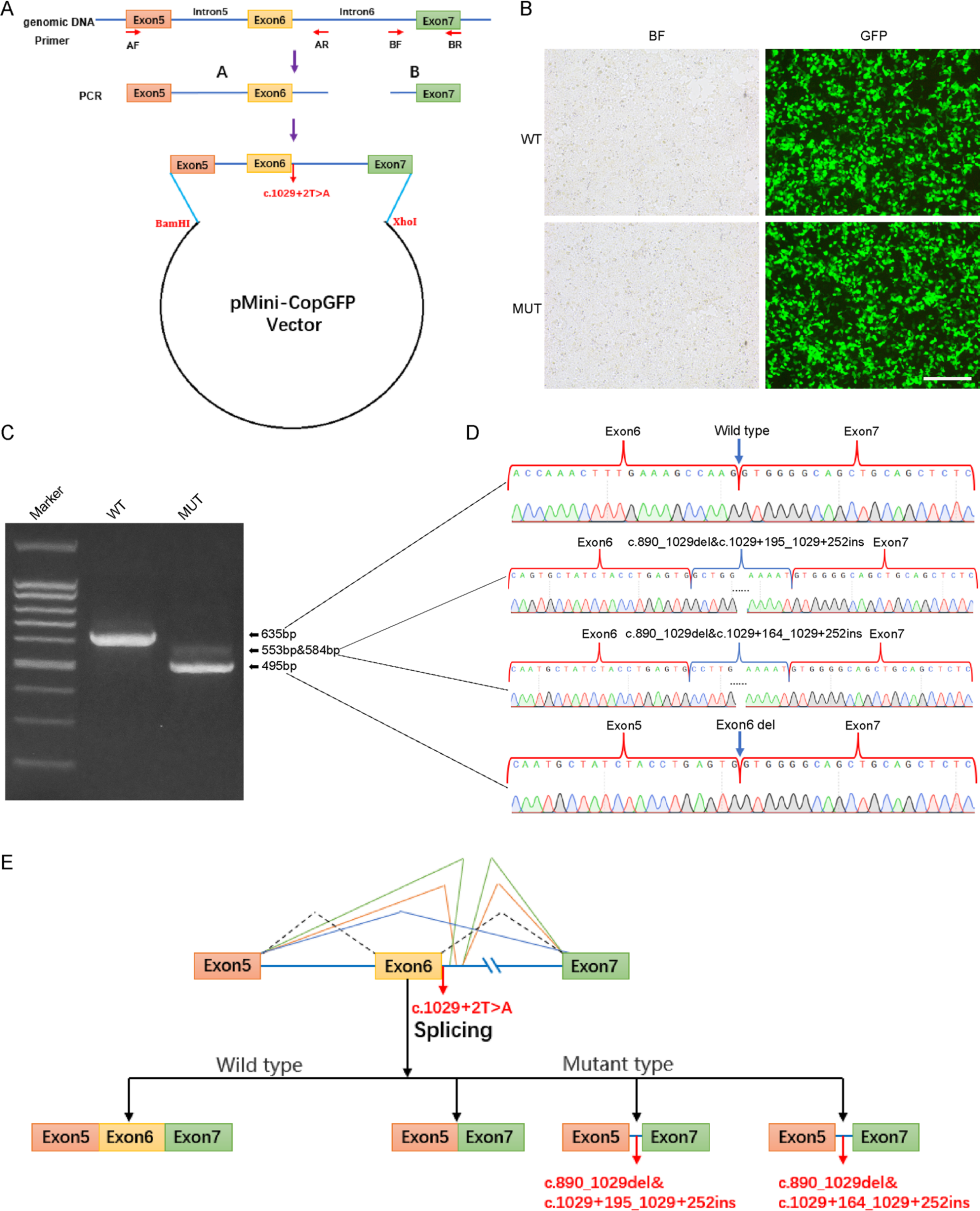
Minigene splicing assay validation of the c.1029 + 2T > A variant. (**A**) Schematic workflow of minigene construct generation. Genomic fragments spanning SERPING1 exons 5–7 with flanking intronic sequences were cloned into the pMini-CopGFP vector. (**B**) Representative bright-field and GFP fluorescence images of HEK293T cells 48 h post-transfection with wild-type or mutant (c.1029 + 2T > A) constructs. (**C**) Agarose gel electrophoresis of RT-PCR products from transfected cells. (**D**) Sanger sequencing chromatograms identifying the canonical transcript and three aberrant splice products. (**E**) Schematic diagram illustrating splicing patterns from the minigene assay

To confirm the causal relationship between the c.1029 + 2T > A variant and the observed splicing defect, we conducted a minigene splicing assay. Genomic fragments containing exons 5 through 7, along with their flanking intronic sequences, were cloned into the pMini-CopGFP reporter vector, incorporating either the wild-type or the c.1029 + 2T > A mutant allele (Fig. 2A). After transient transfection of HEK293T cells (Fig. 2B), RT-PCR analysis of the transcripts derived from the minigene was performed. The wild-type construct generated a single correctly spliced product, while the mutant construct failed to produce a detectable canonical transcript. Instead, three aberrant splice isoforms were identified: (1) c.890_1029del, which corresponds to the complete skipping of exon 6 and represents the predominant aberrant transcript, mirroring the splicing defect observed in patient-derived cells; (2) c.[890_1029del; 1029+195_1029 + 252ins], predicted to result in an in-frame deletion (p.Phe355_Glu362del); and (3) c.[890_1029del; 1029+164_1029 + 252ins], predicted to encode an altered protein segment (p.Lys298_Lys343delinsLAKADIVYFRLETGFSSFFMHLYYIEWQN) (Fig. 2C-E). The latter two isoforms were not detected in patient blood, likely due to nonsense-mediated decay (NMD) or low expression levels.

These findings demonstrate that the novel c.1029 + 2T > A variant disrupts the canonical splicing of *SERPING1*, primarily resulting in exon 6 skipping, disruption of the open reading frame, and the production of a prematurely truncated protein. The aberrant transcripts are likely subject to NMD-mediated degradation, ultimately leading to C1-INH haploinsufficiency and functional deficiency, which constitutes the molecular basis for type I hereditary angioedema (HAE) in this patient.

### The treatment of this patient with HAE

Given recurrent edema episodes, we initiated long-term prophylaxis with subcutaneous lanadelumab 300 mg every two weeks on September 16, 2025. Follow-up evaluations until March 13, 2026 (approximately six months post-treatment) showed that the patient did not experience any further episodes of edema, achieving zero attacks. The only adverse event was mild localized stiffness, occurring three days post-injection and resolving spontaneously. The Angioedema Control Test (AECT) score improved from a baseline of 9.6 to 16. At the final follow-up, C4 and C1-INH levels were not re-evaluated at final follow-up given excellent disease control. Given the current status of disease control and existing guideline recommendations, the decision was made to continue the original Lanadelumab treatment regimen.

### A retrospective study of HAE cases at a single center

**Fig. 3:**
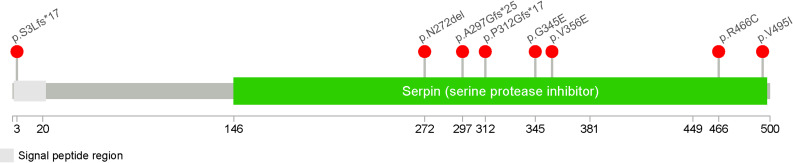
Distribution of SERPING1 variants across functional protein domains

We retrospectively analyzed seven HAE cases at our center (Fig. 3; Table 1). Age at diagnosis ranged from 16 to 43 years (5 females, 2 males). All patients experienced recurrent episodes of edema affecting various anatomical sites, including the face, limbs, buttocks, and abdomen. Diagnostic delay ranged up to 20 years, with 3 patients initially misdiagnosed with allergic condition. Three patients had a family history of HAE. Attack frequency ranged from 1 to 14 episodes annually, with episodes lasting 1–5 days. Laboratory assessments focused on measuring C4 levels, C1 inhibitor (C1-INH) concentration, and C1-INH functionality. Six patients had reduced C4 levels, C1-INH concentration, and C1-INH function. Case #4 showed reduced C4 and C1-INH function with elevated C1-INH concentration. Consequently, Case #4 was diagnosed with type II HAE; the others had type I HAE.

**Table 1:** Diagnosis and treatment of HAE cases: a retrospective review

	Case#1	Case#2	Case#3	Case#4	Case#5	Case#6	Case#7
Sex	Female	Female	Female	Female	Male	Female	Male
Age at diagnosis (years)	43	34	17	36	34	16	24
Age at first onset (years)	39	30	17	16	26	16	22
Diagnostic delay (years)	4	4	-	20	8	-	2
Family history (Y/N)	N	N	N	Y	Y	Y	N
Anatomic sites	• Face • Extremities • Larynx	Left palm and left upper limb	•Face	• Face • Extremities • Larynx • Abdomen	• Buttocks • Extremities	Left palm	Extremities
Frequency per year	13	4	1	14	8	1	5
Duration (days)	3–5	2–5	2–5	2–5	2–3	5	1–2
C4 (g/L)	0.02	0.05	0.03	0.03	0.02	0.04	0.03
C1-INH	0.02 g/L	0.08 g/L	25.78ug/ml	0.7 g/L	0.07 g/L	14.98ug/ml	0.11 g/L
C1INH function	13.1%	21.4%	7%	6.3%	4.3%	7%	2.5%
HAE type	I	1	1	Ⅱ	1	1	1
History of misdiagnosis (Y/N)	Y	N	N	Y	Y	N	N
Misdiagnosis as (disease)	Allergy	-	-	Allergy	Allergy	-	-
Coding region variant	c.1483G > A	c.816_818delCAA	c.6dup	c.1396 C > T	c.1067T > A	c.933_934insGGAA	c.1034G > A
Location	Exon8	Exon5	Exon2	Exon8	Exon7	Exon6	Exon7
Amino acid change	p.Val495Ile	p.Asn272del	p.Ser3Leufs*17	p.Arg466Cys	p.Val356Glu	p.Pro312Glyfs*17	p.Gly345Glu
ACMG classification	VUS	VUS	LP	VUS	VUS	LP	VUS
Literature report	[21]	[22]	[25]	[23, 24]	[1]	[26]	[21]
Severity	Mild	Mild	Moderate	Severe (requiring bed rest)	Variable	Moderate	Mild
LTP received (Y/N)	Y	Y	Y	Y	Y	N	N
LTP agent	lanadelumab	lanadelumab	lanadelumab	lanadelumab	lanadelumab	-	-
Dose & frequency	300 mg q2w	300 mg q2w	300 mg q2w	300 mg q2w	300 mg q2w	-	-
Long term Follow‑up	12-month	17-month	27-month	12-month	20-month	-	-
Outcome	zero attacks	zero attacks	zero attacks	zero attacks	one attack		

*SERPING1* mutations are the primary genetic cause of HAE types I and II. We identified *SERPING1* variants in all seven patients, including three patients with insertions, deletions, or duplications. These mutations typically cause frameshift and premature termination. Four patients had missense mutations. Notably, Two mutations localized to exon 8, which encodes the reactive center loop (RCL), a mutation hotspot in HAE. Although exon 8 missense mutations typically cause HAE type II, the patient with c.1396 C > T had HAE type II while the patient with c.1483G > A had HAE type I. All variants have been previously reported as pathogenic [1, 21–26].

Four patients received no acute treatment, while three patients received on-demand ecallantide 30 mg. ive patients received long-term prophylactic (LTP) therapy, with subcutaneous lanadelumab 300 mg every 2 weeks, later adjusted to every 4–8 weeks. As of March 2026, follow-up duration ranged from 12-27months, showed significant attack reduction in all five patients. Four patients achieved zero attacks; one had a single attack. These findings demonstrate lanadelumab efficacy as LTP for HAE.

## Discussion

We identified and functionally characterized a novel *SERPING1* splice-site variant, c.1029 + 2T > A, that causes type I HAE through exon 6 skipping. These findings expand the mutational spectrum of *SERPING1* and provide insights into the molecular mechanisms underlying HAE, while our clinical data demonstrate the efficacy of Lanadelumab as long-term prophylaxis in Chinese HAE patients.

### Novel *SERPING1* variants and functional characterization

The c.1029 + 2T > A variant disrupts the canonical GT splice donor site of intron 6, resulting predominantly in exon 6 skipping. Both patient-derived RNA analysis and minigene assays demonstrated that this variant abolishes normal splicing, leading to a frameshift and premature termination (p.Ala297Glyfs*25). The resulting truncated protein is likely subject to nonsense-mediated decay, causing C1-INH haploinsufficiency characteristic of type I HAE. Splice-site variants account for approximately 10–15% of all *SERPING1* mutations and frequently result in exon skipping or cryptic splice site activation [10, 14]. Our functional validation approach, combining patient RNA analysis with minigene assays, provides definitive evidence of pathogenicity and represents a robust methodology for characterizing variants of uncertain significance.

### *SERPING1* mutational spectrum and genotype-phenotype correlations

SERPING1 variants are distributed across all eight exons with notable clustering in exon 8, which encodes the reactive center loop [9, 10, 27]. Consistent with literature, four of seven variants (57%) localized to exon 8, reflecting the functional importance of this domain. Missense mutations in the RCL typically result in type II HAE by producing dysfunctional protein, whereas nonsense, frameshift, and splice-site mutations predominantly cause type I HAE through reduced protein levels. However, exceptions exist, as demonstrated by our case with the c.1483G > A missense mutation presenting as type I HAE, likely due to impaired protein folding or secretion.

Genotype-phenotype correlations in HAE remain incompletely defined. While some studies suggest that type II HAE may be associated with earlier onset and more severe symptoms, others report no significant differences in clinical severity between types I and II [28]. In our cohort, attack frequency ranged from 1 to 14 episodes annually regardless of mutation type, emphasizing that clinical presentation alone cannot reliably predict the underlying genetic defect. The absence of family history in 57% of our cases further complicates early diagnosis and underscore the importance of maintaining clinical suspicion even without familial clustering.

### Lanadelumab as first-line long-term prophylaxis in China

Lanadelumab, a fully human monoclonal antibody targeting plasma kallikrein, was approved in China in 2021 and is recommended as first-line long-term prophylaxis in the Chinese HAE management guidelines. A real-world study conducted in China demonstrated that lanadelumab significantly reduced various disease-related indicators in six HAE patients: reducing attack rate by 97.8%, attacks requiring treatment by 98.5%, and work absenteeism by 98.9%, suggesting potential for zero attacks and life normalization [29].

Our data provide important real-world evidence for Lanadelumab efficacy in Chinese HAE patients. Zero attacks were achieved in five of six patients receiving long-term prophylaxis, and our index case achieved zero attacks over six months with only mild self-limiting injection site reactions. These results are consistent with international reports and support lanadelumab as first-line prophylaxis in the Chinese population. AECT score improvement (9.6 to 16) reflects meaningful disease control enhancement.

Regarding safety, lanadelumab has demonstrated an excellent tolerability profile in clinical trials and real-world studies, with injection site reactions being the most common adverse event, occurring in approximately 30–40% of patients but rarely leading to discontinuation. Serious adverse events are rare, and no significant immunogenicity concerns have been identified [17]. Limited data in Chinese patients suggest similar safety. Lanadelumab was well-tolerated in 50 patients, with injection site reactions in 42% and no treatment discontinuations due to adverse events [30]. Our findings align, with only transient injection site stiffness observed.

### Diagnostic challenges and the case for routine *SERPING1* screening

Diagnostic delay (3.9–26 years globally) was mirrored in our cohort (up to 20 years) and three of seven patients (43%) were initially misdiagnosed [31, 32]. This delay results in unnecessary suffering, inappropriate treatments, and increased risk of life-threatening laryngeal edema. Clinical overlap with common angioedema and low disease awareness contribute to diagnostic challenges.

Given severe consequences of delayed diagnosis and availability of effective therapies, SERPING1 genetic testing and C1-INH screening should be incorporated into routine workup of patients with recurrent angioedema particularly when the following features are present: (1) angioedema without urticaria; (2) lack of response to antihistamines and corticosteroids; (3) recurrent abdominal pain episodes; (4) family history of similar symptoms; or (5) onset in adolescence or early adulthood. A tiered diagnostic approach could be implemented, beginning with complement C4 screening as an inexpensive and sensitive initial test (low C4 in > 95% of HAE type I/II patients during attacks and often between attacks), followed by C1-INH antigen and functional assays, and finally genetic confirmation through *SERPING1* sequencing.

The cost-effectiveness of such screening must be weighed against the costs of misdiagnosis, including unnecessary emergency department visits, hospitalizations, and the risk of fatal laryngeal edema. Decreasing costs make routine *SERPING1* screening increasingly feasible. Early genetic diagnosis enables presymptomatic identification of at-risk family members, allowing genetic counseling and proactive management.

In conclusion, our study expands the mutational spectrum of *SERPING1*, provides functional validation of a splice-site mutation through complementary methodologies, and contributes real-world evidence for lanadelumab efficacy and safety in Chinese HAE patients. These findings support the integration of comprehensive genetic testing into HAE diagnostic algorithms and reinforce the role of lanadelumab as first-line long-term prophylaxis. Enhanced HAE awareness among clinicians and broader implementation of C1-INH and *SERPING1* screening could significantly reduce diagnostic delays and improve outcomes in this underdiagnosed population.

## Supplementary Information

Below is the link to the electronic supplementary material.

Supplementary Material 1

## Data Availability

No datasets were generated or analysed during the current study.
