# Supplementary material for: A novel SERPING1 splice-site variant (c.1029 + 2T > A) causing hereditary angioedema type I: functional characterization and clinical analysis
Source: Orphanet J Rare Dis. 2026 Jun 2;21:271. doi: 10.1186/s13023-026-04421-3 (PMC13455203; doi:10.1186/s13023-026-04421-3)
Supplement: Supplementary file 1 [file 13023_2026_4421_MOESM1_ESM.docx]

Figure 1D

Figure 2C
